# Adherence to Antiretroviral Therapy and Associated Factors Among People Living With HIV at the Yaounde Military Hospital, Cameroon: A Cross‐Sectional Study

**DOI:** 10.1155/arat/8888985

**Published:** 2026-06-28

**Authors:** Ethel S. Shang, Clovis Nkoke, Julius Nwobegahay, Charles Njumkeng, Betrand A. Tambe

**Affiliations:** ^1^ Department of Public Health and Hygiene, University of Buea, Buea, South West Region, Cameroon, ubuea.cm; ^2^ Department of Internal Medicine, University of Buea, Buea, South West Region, Cameroon, ubuea.cm; ^3^ Military Health Research Center, CRESAR, Yaounde, Centre Region, Cameroon; ^4^ Division Human Nutrition, Stellenbosch University, Cape Town, South Africa, sun.ac.za

**Keywords:** adherence, antiretroviral therapy, pill count, PLHIV, viral suppression

## Abstract

**Background:**

Adherence to antiretroviral therapy (ART) is an essential component in the global response to HIV/AIDS. Despite increased access to ART in Cameroon, maintaining adherence to ART remains a significant challenge, jeopardising progress toward the global 95‐95‐95 HIV treatment targets.

**Objectives:**

To assess the level of adherence to ART and associated factors among people living with HIV at Yaounde Military Hospital in Cameroon.

**Methods:**

A cross‐sectional study was conducted from January to June 2025 among people living with HIV (PLHIV) aged 10 years and above receiving ART at the Yaounde Military Hospital. A simple random sampling technique was employed, whereby all eligible participants presenting for ART refill during the study period were invited to participate. A total of 356 PLHIV were interviewed using a structured questionnaire. Adherence to ART was measured using pill count and viral suppression. Descriptive statistics summarised demographic and clinical variables, Chi‐square tests assessed associations between categorical variables, and logistic regression identified factors associated with adherence. Statistical significance was set at *p* < 0.05.

**Results:**

The mean age of participants was 46.5 ± 15.1 years, ranging from 10 to 84 years, and the mean duration on ART was 9.4 ± 5.8 years, ranging from 0 to 24 years. An overall adherence rate, measured by pill count, was 86.0% (95% CI: 82.35–89.57), while out of 356 participants, 344 (96.6%; 95% CI: 94.7–98.3) were virally suppressed. Factors significantly associated with adherence included smoking (AOR = 5.27, 95% CI = 1.72–16.13, *p* = 0.004), male gender (AOR = 2.8, 95% CI = 1.12–7.31, *p* = 0.027) and ART adverse effects (AOR = 0.35, 95% CI = 0.14–0.87, *p* = 0.023).

**Conclusion:**

ART adherence in this study fell below the UNAIDS optimal target of ≥ 95%. Factors such as smoking, male gender and ART adverse effects significantly influence adherence. Targeted interventions addressing these barriers are essential to optimise adherence and sustain progress toward the 95‐95‐95 HIV treatment targets in Cameroon.

## 1. Introduction

HIV infection remains a major public health challenge, with sub‐Saharan Africa bearing the greatest burden. Nevertheless, a global commitment exists to halt new HIV infections and ensure access to treatment for PLHIV [1]. According to 2024 UNAIDS estimates, approximately 40.8 million people were living with HIV globally, including 39.4 million adults and 1.4 million children under 15. New infections declined by 40% since 2010, totalling 1.3 million in 2024 [2]. Eastern and Southern Africa account for the highest number of people living with HIV (PLHIV) at 21.1 million, followed by Western and Central Africa (5.2 million) [3].

In Cameroon, adult HIV prevalence (ages 15–49 years) was estimated at 2.6% in 2023, with about 20,000 new infections in 2024. The 2024 prevalence (2.3%) remains among the highest in West and Central Africa [4]. Despite substantial national efforts to expand antiretroviral therapy (ART), testing and prevention, long‐term disease management remains challenging.

The UNAIDS 95‐95‐95 targets aim for 95% of PLHIV to know their status, 95% of diagnosed individuals to be on sustained ART and 95% of those on ART to achieve viral suppression by 2030 [5]. The second target underscores the importance of initiating and retaining PLHIV on lifelong ART, a critical step for preventing disease progression, reducing mortality and curbing transmission [6]. Globally, 87% of PLHIV know their status; among these, 77% receive ART and 73% achieve viral suppression. Several sub‐Saharan African countries have already met the 95‐95‐95 targets [5]. Cameroon is progressing, with 92% aware of their status, 88% on treatment and 82% virally suppressed [7].

Adherence to ART defined as taking 95%–100% of medications as prescribed is essential for viral suppression and preventing drug resistance [8]. However, maintaining this level is difficult in resource‐limited settings due to food insecurity, transportation barriers, side effects and stigma [9]. Nonadherence leads to treatment failure, resistant strains and higher healthcare costs [10]. While East and Southern Africa have achieved 80% ART coverage, West and Central Africa lag at 45% [11].

In Cameroon, despite ART scale‐up, suboptimal adherence undermines treatment outcomes. Although the country is advancing toward the UNAIDS targets, adherence below recommended thresholds compromises viral suppression, fuels drug resistance and sustains HIV transmission [12, 13]. Socioeconomic constraints, stigma, medication side effects and health system barriers negatively influence adherence. At the Yaoundé Military Hospital, structural and occupational factors may uniquely shape adherence behaviours, yet evidence remains scarce. Therefore, this study aims to assess adherence levels and associated factors among PLHIV at the Yaoundé Military Hospital, Cameroon, to generate evidence that can inform targeted interventions and improve treatment outcomes.

## 2. Methods

### 2.1. Study Design

The cross‐sectional study was conducted in the Yaounde Military Hospital in the Centre region of Cameroon.

### 2.2. Study Setting

This study was conducted in the Yaounde Military Hospitals (YMH) in Cameroon, a sub‐Saharan African country with a high HIV prevalence [14], hosting a population of over 28 million inhabitants divided into 10 regions, bordered by Nigeria, Chad, Central African Republic, Gabon, Equatorial Guinea and Congo. Specifically, the study took place at the YMH in the Centre region. This hospital caters for a diverse population, including military personnel, their families and civilians, and offers comprehensive HIV/AIDS care and treatment services such as counselling and support services, making them ideal locations for researching HIV treatment and care. This centre was purposefully selected based on the number of PLHIV registered in the centre, allowing for efficient recruitment of the required sample.

### 2.3. Study Population

The study included PLHIV of both sexes and aged 10 years and above, on HIV treatment for ≥ 6 months, with at least 1 clinic visit between February and March 2025, and who provided informed consent or assent. All PLHIV who presented for monthly ART refill within the data collection period and met the inclusion criteria were invited to participate. Critically ill PLHIV who were unable to respond to the questionnaire were excluded from the study.

### 2.4. Data Collection/Measurement

A face‐to‐face interview with PLHIV by trained interviewers at the HIV/AIDS treatment centre was conducted using a structured questionnaire. The questionnaire was entered into the kobo tool box. Viral load was extracted from clinical records. Participants were asked to bring their medication to the treatment centre, where the number of remaining pills in the medication container was counted.

Adherence to treatment was measured using pill (with participants classified as adherent if they took ≥ 95% of prescribed pills and nonadherent if < 95%) and viral load suppression (< 1000 copies/mL). Due to variability in refill intervals ranging from monthly to multimonth dispensing under differentiated service delivery models, the adherence period was not uniform across participants. To account for this inconsistency, adherence was calculated using the formula

(1) Adherence %=Pills dispensed−Pills returnedPrescribed daily dose × Number of days between visits×100.

For viral load, the most recent viral load results of the participants were used and those with fewer than 1000 copies per millilitre were classified as virally suppressed.

### 2.5. Sample Size

The sample size was determined using Cochran’s formula: *n* = (*Z* ^2^ *p*(1 − *p*))/*e* ^2^, where *n* = minimum sample size, *z* = 1.96 with 95% CI, *e* = margin of error and *p* = percentage of adherence (77.5%). This was done using the following assumptions:

Confidence interval (CI) of 95% with standard normal variation (*Z*) of 1.96 and margin of error (*e*) being 5%.

The adherence rate from a similar study (*P*) done in Yaounde Central Hospital was 77.5% [99].

*n* = (〖1.96〗^2^ 0.775 (1–0.775))/〖0.05〗^2^  = 267.94 participants.

The minimum required sample size for this study was calculated to be 267.94 based on the desired confidence level, margin of error and estimated effect size. However, to strengthen the reliability of the results and account for potential nonresponses, incomplete data and unforeseen exclusions, an additional 32.9% was added to the calculated sample size. This adjustment resulted in a final sample size of 356 participants. The increased sample size was intended to enhance the statistical power and generalisability of the study findings.

### 2.6. Sampling Technique

Participants were selected using a simple random sampling technique from a list of all eligible PLHIV scheduled for ART refill during the study period. The sampling frame consisted of all PLHIV at the Yaoundé Military Hospital. Each eligible individual on the list was assigned a unique identifier retrieved from the facility’s treatment register, and study participants were selected by drawing these codes using random number generator to obtain the sample size.

### 2.7. Data Analysis

Information from the forms was checked each time it was filled to ensure completeness at the point of collection. Data collected were extracted to an Excel sheet and analysed using statistical software SPSS Version 26. Electronic data were stored securely in capture tools such as Kobo tool box, and regular data quality checks were performed. Participants’ demographics and outcome variables were summarised using descriptive statistics. Continuous variables were presented as mean and standard deviation, while categorical variables were summarised using frequencies and percentages.

The outcome variable was adherence to antiretroviral treatment. Independent variables included age group, sex, marital status, education level, employment status, religion, smoking status, ART reminder use, ART‐related adverse effects and duration on ART. Adherence to treatment was measured using pill count (adherent: ≥ 95% of pills taken, nonadherent: < 95% of pills taken) and viral load suppression (< 1000 copies/mL). Chi‐square tests were used to assess the relationships between categorical variables. Logistic regression was used to identify factors associated with adherence and other variables such as age groups, sex and education levels. Subgroup analyses were conducted using bivariate and multivariable logistic regression across demographic and clinical characteristic. Variables with a *p* value < 0.2 at the bivariate proceeded to the multivariate analysis to avoid excluding potentially important variables that may become significant after adjustment for confounders. Statistical significance was set at *p* value < 0.05.

## 3. Results

### 3.1. Sociodemographic Characteristics of the Study Population

A total of 356 participants were enrolled and included in the analysis. Participants ages range from 10 to 84 years with mean age of 46.5 (SD 15.1) years. A majority of the participants 132 (37.1%) were within the age range of 35–49 years. Females were the most respected sex of the study population, 256 (71.9%). Regarding educational level, most participants, 200 (56.2%), had attended secondary education (Table 1). Most participants, 196(55.1%), were Catholic. In terms of marital status, 144 (40.0%) were single, 102 (28.7%) married and 58(16.3%) were cohabiting.

**TABLE 1:** Description of the study population at the YMH.

Variable		Frequency	Percentage
Sex	Male	100	28.1
	Female	256	71.9
	Total	356	100

Age group (years)	10–19	28	7.9
	20–34	36	10.1
	35–49	132	37.1
	50–64	124	34.8
	> 65	36	10.1
	Total	356	100

Religion	None	32	9.0
	Catholic	196	55.1
	Protestant	78	21.9
	Pentecostal	44	12.4
	Muslim	6	1.7
	Total	356	100

Employment status	Employed	214	60.1
	Unemployed	142	39.9
	Total	356	100

Level of education	No formal education	12	3.4
	Primary	88	24.7
	Secondary	200	56.2
	Tertiary	56	15.7
	Total	356	100

Marital status	Single	144	40.0
	Co‐habitation	58	16.3
	Divorced	10	2.8
	Married	102	28.7
	Widow/widowed	42	11.8
	Total	356	100

### 3.2. Clinical Characteristics of the Study Population

From the clinical characteristics, it was observed that most participants did not report ART adverse effects, 270 (75.8%), and a large proportion, 264 (74.2%), did not use any ART reminder. Caregiver satisfaction was generally high, with 238 (66.9%) reporting full satisfaction. Nearly all participants, 352 (98.9%), were on multimonth ART dispensation, and 260 (73.0%) were diagnosed with HIV more than five years ago. Most participants, 306 (86.0%), were on a once‐daily single‐tablet regimen, and 350 (98.3%) were receiving first‐line treatment. Participants’ duration on treatment ranged from months to 24 years, with a mean duration on ART of 9.4 (5.8) years. As concerns smoking status, a majority, 328 (892.1%), were nonsmokers as compared to 28 (7.9%) who were smokers (Table 2).

**TABLE 2:** Clinical factors of the study population at the YMH.

Variable		Frequency	Percentage
ART adverse effects	No	270	75.8
	Yes	86	24.2
	Total	356	100

ART reminder	No	264	74.2
	Yes	92	25.8
	Total	356	100

Caregiver satisfaction	Partial	118	33.1
	Full	238	66.9
	Total	356	100

ART dispensation model	Single month	4	1.1
	Multimonth	352	98.9
	Total	356	100

Duration since diagnosis	< 1 year	4	1.1
	1–5 years	92	25.8
	> 5 years	260	73.0
	Total	356	100

Physical activity	No	128	36.0
	Yes	228	64.0
	Total	356	100

Smoking status	Nonsmokers	328	92.1
	Smokers	28	7.9
	Total	356	100

Number of ARV tablets consumed per day	1	306	86.0
	2	44	12.4
	3	4	1.1
	4	2	0.6
	Total	356	100

Duration on ART (years)	≤ 5	104	29.2
	6–10	120	33.7
	> 10	132	37.1
	Total	356	100

Treatment regimen	First line	350	98.3
	Second line	6	1.7
	Total	356	100

### 3.3. Level of Adherence to ART Among PLHIV at the Yaounde Military Hospital

**FIGURE 1:**
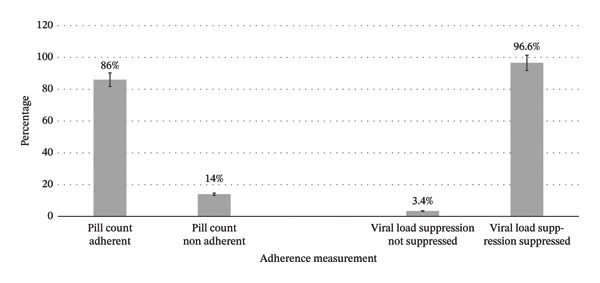
Level of ART adherence among PLHIV attending the YMH.

Out of the 356 participants assessed, 306 (86.0%; 95% CI: 82.35–89.57) were classified as adherent based on pill count. Additionally, viral load data indicated that 344 participants, 96.6% (95% CI: 94.7–98.3), were virally suppressed (Figure 1).

### 3.4. Factors Associated With ART Adherence Among PLHIV at the Yaounde Military Hospital

#### 3.4.1. Sociodemographic Factors Associated With ART Adherence

The bivariate analysis revealed several significant associated sociodemographic factors with adherence to ART. It was observed that male participants were more likely to be adherent compared to females (COR = 1.09, 95% CI: 1.01–1.18, *p* = 0.039). On the other hand, it was observed that the probability of adherence was about 5% higher among participants with secondary education (COR = 0.88, 95% CI: 0.78–0.98, *p* = 0.024). The probability of being adherent was 17% higher among nonsmokers compared to smokers (COR = 1.17, 95% CI: 1.03–1.34, *p* = 0.020). Other factors such as age, marital status, employment, religion and duration on ART did not show any associations (Table 3). After adjusting for confounders, two factors remained statistically significant. The results showed that participants who smoke are five times more likely to be adherent compared to those who do not smoke (AOR = 5.27, 95% CI: 1.72–16.13, *p* = 0.004). In addition, male participants were three times more likely to be adherent compared to females (AOR = 2.8, 95% CI: 1.12–7.31, *p* = 0.027).

**TABLE 3:** Sociodemographic factors associated with ART adherence among PLHIV at the YMH.

Variables	Level of adherence		COR	95% confidence interval		*p* value	AOR	95% confidence interval		*p* value
	Nonadherent *n* (%)	Adherent *n* (%)								
Age group (years)										
10–19	2 (7.1)	26 (92.9)	1.16	0.98	1.38	**0.082** ^**∗**^	4.16	0.44	25.07	0.118
20–34	8 (22.2)	28 (77.8)	1.00	0.85	1.17	1.000	0.52	0.19	3.230	0.325
35–49	18 (13.6)	114 (86.4)	1.09	0.96	1.24	**0.19** ^**∗**^	2.14	0.90	8.43	0.154
50–64	14 (11.3)	110 (88.7)	1.12	0.98	1.27	**0.094** ^**∗**^	2.28	0.92	8.13	0.129
65–85	8 (22.2)	28 (77.8)	1	—	—	—	1	—	—	—
Sex										
Male	8 (8.0)	92 (92.0)	1.09	1.01	1.18	**0.039** ^**∗**^	2.87	1.12	7.31	**0.027** ^**∗**^
Female	42 (16.4)	214 (83.6)	1	—	—	—	1	—	—	—
Marital status										
Single	26 (18.1)	118 (81.9)	0.92	0.82	1.03	**0.159**				
Co‐habitation	8 (13.8)	50 (81.9)	0.96	0.84	1.10	0.542				
Divorced	2 (20.0)	8 (80.0)	0.90	0.71	1.14	0.389				
Married	10 (9.8)	92 (90.2)	0.99	0.88	1.13	0.965				
Widow/widower	4 (9.5)	38 (90.55)	1	—	—	—				
Education										
No formal education	2 (16.7)	10 (83.3)	0.91	0.73	1.13	0.385	0.47	0.74	3.03	0.429
Primary	18 (20.5)	70 (79.5)	0.88	0.78	0.98	**0.024** ^**∗**^	0.33	0.10	1.05	**0.061** ^**∗**^
Secondary	26 (33.1)	166 (87.4)	0.94	0.85	1.05	0.261	0.56	0.18	1.75	0.319
Tertiary	4 (7.1)	52 (92.9)	1	—	—	—	—	—	—	—
Place of worship										
None	2 (6.3)	30 (93.8)	1.31	0.97	1.77	**0.08** ^**∗**^	6.90	0.71	66.98	0.960
Catholic	24 (12.2)	172 (87.8)	1.24	0.94	1.63	**0.14** ^**∗**^	3.87	0.64	23.60	0.142
Protestant	10 (12.8)	68 (87.2)	1.23	0.92	1.63	**0.157** ^**∗**^	4.78	0.72	31.95	0.107
Pentecostal	12 (27.3)	32 (72.7)	1.06	0.79	1.42	0.68	1.17	0.18	7.66	0.873
Mosque	2 (33.3)	4 (66.7)					—	—	—	—
Employment status										
Employed	34 (15.9)	180 (84.1)	0.96	0.89	1.03	0.218				
Unemployed	16 (11.3)	126 (88.7)	1	—	—	—				
Smoking status										
Nonsmoker	42 (12.8)	286 (87.2)	1.17	1.03	1.34	**0.020** ^**∗**^	5.27	1.72	16.13	**0.004** ^**∗**^
Smoker	8 (28.6)	20 (71.4)	1	—	—	—	1	—	—	—
Alcohol consumption										
No	16 (11.9)	118 (88.1)	1.03	0.96	1.11	0.37				
Yes	34 (15.3)	188 (84.7)	1	—	—	—				
ART reminder										
No	42 (15.9)	222 (84.1)	0.93	0.86	1.01	**0.085** ^**∗**^	0.62	0.25	1.55	0.309
Yes	8 (8.7)	84 (91.3)	1	—	—	—				

*Note:* The bold values indicate that the variables are significant at *p* < 0.05.

^∗^Variables significant at *p* < 0.05.

#### 3.4.2. Clinical Factors Associated With ART Adherence Among PLHIV at the YMH

Table 4 presents bivariate (COR) and multivariate (AOR) logistic regression analyses for clinical factors. In the bivariate analysis, only duration on ART of 6–10 years showed a borderline association with lower adherence compared to > 10 years (COR = 0.92, *p* = 0.070). No other clinical factors were statistically significant. After adjusting for confounders in the multivariate model, only ART adverse effects were significant. Participants who reported no adverse effects were 65% less likely to adhere compared to those who experienced adverse effects (AOR = 0.35, 95% CI: 0.14–0.87, *p* = 0.023).

**TABLE 4:** Clinical factors associated with ART adherence among PLHIV at the YMH.

Variables	Level of adherence		COR	95% confidence interval		*p* value	AOR	95% confidence interval		*p* value
	Nonadherent *n* (%)	Adherent *n* (%)								
ART adverse effects										
No	42 (15.6)	228 (84.4)	0.94	0.86	1.02	**0.145** ^**∗**^	0.35	0.14	0.87	**0.023** ^**∗**^
Yes	8 (9.3)	78 (90.7)	1	—	—	—	1	—	—	—
Duration on ART (years)										
≤ 5	10 (9.6)	94 (90.4)	1.03	0.98	1.12	0.579				
6–10	24 (20.0)	96 (80.0)	0.92	0.85	1.01	**0.070** ^**∗**^				
> 10	16 (12.1)	116 (87.9)	1	—	—	—				
Dispensation model										
Single month	0 (0.0)	4 (100)	1.15	0.82	1.62	0.42				
Multimonth	50 (14.2)	302 (85.8)	1	—	—	—				
Care services										
Average	2 (33.3)	4 (66.7)	0.84	0.22	0.63	1.11				
Good	12 (9.8)	110 (90.2)	1.06	**0.12**	0.98	1.15				
Excellent	36 (15.8)	192 (84.2)	1	—	—	—				
Caregiver satisfaction										
Partial	14 (11.9)	104 (88.1)	1.03	0.40	0.96	1.12				
Full	36 (15.1)	202 (84.9)	1	—	—	—				
Line of treatment										
First line	50 (14.3)	300 (85.7)	0.87	0.32	0.66	1.15				
Second line	0 (0.0)	6 (100)	1	—						
Duration since diagnosis										
< 1 year	0 (0.0)	4 (100)	1.17	0.38	0.83	1.64				
1–5 years	10 (0.9)	82 (89.1)	1.05	0.28	0.96	1.14				
> 5 years	40 (15.4)	220 (84.6)	1	—	—	—				

*Note:* The bold values indicate that the variables are significant at *p* < 0.05.

^∗^Variables significant at *p* < 0.05.

## 4. Discussion

This study assessed adherence to ART and its associated factors among PLHIV at the Yaounde Military Hospital. The study employed a cross‐sectional design to provide valuable insights into ART adherence among PLHIV receiving care in Yaounde Military Hospital.

The findings of this study revealed an adherence level of 86.0% as measured by pill count. This finding aligns with a previous study in Yaounde, which also found high adherence levels and recommended drug level monitoring as a more reliable adherence measure than self‐report [15]. The adherence levels observed in this study was higher than those reported in several other regions of Cameroon, where adherence levels ranged from 50.9% reported in the North region of the country [44] to 62.35% in the North West, Littoral and South West regions [16]. However, another study conducted in Yaounde reported a higher adherence level of 97.5% [14], indicating very high compliance with prescribed ART regimens, which is crucial for effective disease management and improved health outcomes. The difference observed across studies may be attributed to the variations in healthcare access, literacy levels and availability of patient support systems between the study sites.

The adherence level in the current study is also comparable to findings from Northern Egypt, where Magdy et al. reported an adherence rate of 86.0% [17]. In contrast, lower adherence levels of 80% were reported in Harare, Zimbabwe, by Mtisi et al. [18]. These variations across settings may be attributed to the variations in healthcare access, literacy levels and availability of patient support systems between the study sites. In addition, regional disparities within Cameroon may be influenced by differences in partner‐supported interventions, such as the scale and duration of PEPFAR‐supported HIV programs across regions. However, variables stated here for justifications were not used in the current study.

Three patient‐related factors were significantly associated with ART in this study: smoking, male gender and ART side effects. It was observed that male participants had a better adherence to ART than females. This finding is consistent with studies from Nigeria [6], South Africa [7] and other African settings [19, 20] that reported gender disparities in ART adherence. However, it contrasts with findings from Ethiopia [4] and Uganda [5], where no significant sex differences were observed, as well as studies suggesting that men are more likely to miss doses because of side effects, work commitments and disclosure concerns [21].

The lower adherence observed among women in the present study may be explained by interrelated factors. Women often face greater caregiving responsibilities, higher levels of stigma and an increased risk of depression, all of which may negatively affect adherence [8]. Fear of HIV‐related stigma and disclosure may also discourage women from taking medication consistently or openly. Similarly, studies from Bangladesh identified stigma, inadequate counselling and medication stock‐outs as important barriers to ART adherence [22]. Furthermore, a systematic review by Mukumbang et al. [9] identified female sex as a consistent predictor of nonadherence across multiple settings. In the present study, the military healthcare setting dominated by male, with its regimented routines and integrated follow‐up systems, may have contributed to the better adherence observed among male participants.

ART‐related side effects also found to negatively affect adherence in this study. The discomfort and negative health effect caused by the side effect discourage patients from taking their drugs, as they fear possible future side effects. This finding is in agreement with studies conducted among adolescents in sub‐Saharan Africa where medication side effects were identified as significant barriers to adherence [23, 24].

Furthermore, smokers were less likely to adhere to ART compared to nonsmokers. This could be explained by the fact that smokers are likely to engage in behaviours that disrupt routine and affect consistent medication use. Similar findings were reported in a South African study, which identified substance use as a major contributor to nonadherence [25]. However, the findings differ from those of another study, which suggested that smoking was not associated between smoking and ART adherence among individuals with adherence difficulties [26, 27]. This discrepancy between studies may be related to differences in study populations, settings, methodologies or the time periods during which the studies were conducted.

While the study provides valuable insights into ART adherence among PLHIV, it has several limitations. Firstly, adherence at 86.0% falls below the UNAIDS optimal target of 95%, highlighting the need for further investigation into barriers to adherence. The study did not explore key sociodemographic and structural factors, such as income level, stigma or family support, which are known to influence adherence. Additionally, viral load measurements were obtained from routine clinical records rather than being systematically assessed during the study period, potentially limiting their accuracy as a reflection of participants′ viremia status at the time of the study.

Despite these limitations, the study offers important contributions to the HIV program by informing strategies to improve adherence and measure progress toward epidemic control. The use of multiple adherence assessment methods pill count and viral suppression enhances the validity and reliability of the findings. Furthermore, the inclusion of routine programmatic data from a military healthcare setting provides unique insights into adherence in an understudied population.

## 5. Conclusion

This study revealed suboptimal ART adherence (86.0%) among PLHIV at Yaoundé Military Hospital, falling short of the UNAIDS ≥ 95% target, with smoking, male gender and ART adverse effects identified as key predictors of poor adherence. Sustained engagement in HIV care was associated with improved viral suppression, underscoring the importance of continuous clinical support and the need to develop new interventions taking into account the above‐mentioned factors. Given the cross‐sectional design, we recommend longitudinal studies to better understand adherence patterns, incorporating comprehensive socioeconomic factors, systematic viral load monitoring and mixed‐method approaches to identify barriers and facilitators. Such research would inform development of tailored interventions to bridge the adherence gap and advance progress toward global HIV treatment target.

## Author Contributions

Ethel S. Shang and Betrand A. Tambe conceptualised the idea. The study design was developed collaboratively by Ethel S. Shang, Betrand A. Tambe, Clovis Nkoke, Julius Nwobegahay and Charles Njumkeng. Material preparation and data collection were performed by Ethel S. Shang, Betrand A. Tambe and Clovis Nkoke performed the data analysis and interpreted the data. The initial draft was composed by Ethel S. Shang, Betrand A. Tambe, Charles Njumkeng, Julius Nwobegahay and Clovis Nkoke. All authors contributed to the writing, reviewing and editing of the subsequent versions of the manuscript and/or publication of this article.

## Funding

No funding was received for this study.

## Disclosure

All analyses, findings and interpretations are exclusively those of the authors.

## Ethics Statement

Ethical approval for this study was obtained from the Institutional Review Board of the University of Buea (Ref. No: 2025/1691‐12/UB/SG/IRB/FHS), with administrative authorisation from the Faculty of Health Sciences, University of Buea, and the Director of the Yaoundé Military Health Hospital.

Confidentiality was maintained by assigning identification codes instead of names, and participants were informed of their right to withdraw at any time without consequence. Information collected during the survey was used mainly for the purpose of this study.

## Consent

Written assent was obtained from parent/guardian participants under the age of 18 years, while written consent was obtained from participants aged 18 years and above.

## Conflicts of Interest

The authors declare no conflicts of interest.

## Data Availability

All datasets on which the conclusions of the research rely are presented in this paper. However, raw data are available from the corresponding author upon reasonable request.
